# Experience of nursing students regarding clinical support in the management of TB and HIV patients in a primary healthcare setting: a phenomenological study

**DOI:** 10.11604/pamj.2019.33.209.15819

**Published:** 2019-07-16

**Authors:** Tshegofatso Siphora Mekgoe, Kelebogile Lepedi, Patricia Tshepo Makhutle, Lufuno Makhado, Karabo Madiba, Nokuphila Senamile Nontokozo Langa

**Affiliations:** 1 School of Nursing Sciences, North-West University, Mafikeng Campus, Potchefstroom, South Africa; 2 Research Office, School of Health Sciences, University of Venda, University Road, Thohoyandou, 0950, South Africa

**Keywords:** Clinical learning, clinical practice, clinical support, nursing students, management of TB/HIV, primary health care, preceptorship, supervision, theory-practice integration, North West province

## Abstract

**Introduction:**

management of tuberculosis (TB) and Human Immunodeficiency Virus (HIV) within primary health care (PHC) facilities involve nursing students as part of them integrating theory to practice. Clinical learning for nursing students requires adequate support from the Nursing Education Institution (NEI) and nursing professionals. Given the dearth of literature regarding clinical support for nursing students in the management of TB/HIV in PHC setting, this study is aimed at exploring and describing nursing students' experiences regarding clinical support.

**Methods:**

a phenomenological design was used to explore and describe the experiences of nursing students using an individual, unstructured, in-depth interview. Audio-taped interviews were transcribed verbatim and analysed using Atlas TI software.

**Results:**

themes derived from the study were factors inhibiting clinical support which incorporated shortage of professional nurses (PNs), lack of accompaniment, fear of managing TB/HIV patients and negative attitudes of PNs; outcomes of poor clinical support included inability to integrate TB/HIV theory to practice and lack of confidence among nursing students; nursing students' desired outcomes through clinical support included becoming a competent TB/HIV nurse and the ability to integrate TB/HIV theory to practice; and strategies to strengthen and promote clinical support in TB/HIV management through strengthened occupational health and safety learning, provision of knowledge regarding post-exposure prophylaxis and infection control, and appointed clinical PN for students in each facility.

**Conclusion:**

the development of policies for clinical support, increasing supervision, appointment of clinical preceptors and accompanists in facilities where nursing students are placed would promote clinical learning within the NEI and the production of competent and confident nurses.

## Introduction

The management of Tuberculosis (TB) and Human Immunodeficiency Virus (HIV) patients involves professional nurses (PN), enrolled nurses (EN), enrolled nursing auxiliaries (ENA) and nursing students. The rise of TB and HIV in South Africa increases the level of nursing students' exposure to the dual burden throughout their training. However, TB and HIV management seems to be a huge problem to nursing students during their clinical learning placement. The clinical guidance and support for nursing students had been a challenge for PNs, as PNs are faced with a heavy workload to attend to, such as providing care to many patients and they feel that providing guidance and support to students is an extra workload which is over burdened by understaffing [1]. Furthermore, there seems to be uncertainty about who is responsible for clinical accompaniment, guidance and support of student nurses [2]. Hence, the proposed study seeks to explore nursing students' experiences regarding clinical support in their clinical placement and learning. Clinical teaching and learning is the means by which nursing students apply their theoretical knowledge of nursing into practice through integration of theoretical knowledge and skills in the clinical setting, which becomes the art and science of nursing and to fulfil the main aim of the clinical nursing practice which is for nursing students to be able to gain competency through clinical support [3]. Therefore, it is the responsibility of every professional nurse to make sure that they play an educational role to facilitate development of nursing students personally, clinically and academically [2]. In addition, nursing students had been described not to receive adequate accompaniment, guidance and supervision during their clinical learning and practice due to an extreme shortage of PNs, and when nursing students enter the clinical setting for the purpose of clinical learning and practice, they are regarded as nurses filling gaps where there is a shortage of nurses, in order to push clinical work rather than fulfilling their set objectives. This is true given the increased rate of people living with HIV (PLWH) and TB and nursing students end up having feelings of being restricted by professional nurses from fulfilling their learning tasks or outcomes [2].

The management of TB and HIV patients had been said to be a difficult task for nursing students because they become anxious and afraid [4]. Nursing students develop fear of being infected by TB and HIV while managing TB and HIV patients and this fear has the potential to lead to unnecessary injuries/accidents such as needle pricking and contracting TB, to name a few. Nursing students, like PNs who are managing TB and HIV, require training in Nurse-Initiated Management of ART (NIMART) and Prevention of Mother-To-Child Transmission (PMTCT) during pre-services programmes before they can work as a newly qualified professional nurse [5]. Furthermore, this will capacitate them to manage PLWH & TB confidently and they can be in a position to protect themselves against injuries e.g. needle prick during management of HIV patients. In addition, the Nursing Education Institution (NEI) must ensure that these pre-service programmes are established for student nurses before clinical placement [5]. In the midst of all these challenges, strategies can be put in place namely, are practical evaluations and clinical accompaniment by module facilitator from the NEI who visits the clinical services or facilities in order to assess, evaluate and examine nursing students' ability to perform required skills based on the relevant set objectives [6, 7], and all of these can be successful through the aid of mentorship (which facilitate students' learning in clinical placement and strengthen their professionalism), preceptorship, simulation, and professional nurses supervision and guidance. It had been emphasized that pre-service nursing education should ensure that nursing students meet the standards of quality care and safety in patient care [8]. Another study highlighted that it is of paramount importance that nursing students are tested for HIV and screened for TB before their initial visit to the clinical learning setting or practice [9].

Furthermore, nursing students can safely protect themselves from being infected by employing relevant infection control measures and ascertaining whether they could have contracted TB and HIV before or during their engagement in clinical practice and management of TB and HIV. In addition to this, there are marked inconsistencies and lack of ability or opportunity to give accurate feedback on professional values and behaviour in contrast to clinical skills feedback, either in written comments or individual or group discussion, so if student nurses are not being asked to give feedback regarding their clinical experience and challenges from the clinical setting where they were placed for clinical practise, there cannot be descriptions of or improvements towards the experiences, existing gaps and support to be provided [9]. Given the argument above, it is evident that there is a lack of clinical support towards nursing students through preceptorship, supervision, mentoring and coaching during clinical practice and placement which has the potential to lead to their clinical incompetence post-training. This assertion brings the importance to focus on the management of the dual burden of TB and HIV in the PHC. This is true given all the interventions, strategies and policies (namely NIMART, PMTCT, National TB management guidelines etc.) in place to curb the dual burden, however, the problem still persists. Furthermore, this gives rise to the need to investigate the clinical support received by nursing students during pre-service programmes in the management of TB and HIV within the PHC facilities. There is a dearth of literature regarding the clinical support nursing students receive in the management of TB and HIV within the PHC environment. To integrate theory and practice during clinical practice is a challenge for nursing students. This is explained better by the inability to manage some of the cases that they come across at the clinical practices such as TB and HIV cases. Management of TB and HIV in PHC requires competency and knowledge. To be competent one needs to integrate theory and practice, and this can be done easily through the support from the NEI and the professional nurses on duty. Studies conducted by different researchers show that there is an inadequate support for nursing students during their clinical placement and this is manifested by the incompetency of nurses after completion of their education, especially in the care and management of TB and HIV infected patients. This stimulated the researchers of this paper to study the clinical support for nursing students with regard to the management of TB and HIV patients in PHC and to answer the following questions:*what are the experiences of nursing students regarding clinical support in the management of TB and HIV patients in PHC setting?*; *How can clinical support be strengthened and promoted among nursing students in the clinical environment?* The purpose of this study was to explore and describe the experiences of nursing students regarding clinical support in the management of TB and HIV patients in PHC setting.

## Methods

A qualitative, phenomenological research design was used for this study, which was explorative, descriptive and contextual in nature. Explorative study is merely formative for the purpose of gaining new insights, discovering new ideas and increasing knowledge of phenomena of the experiences of nursing students regarding clinical support in the management of TB and HIV in PHC setting and a descriptive study aims at providing an accurate and precise description of experiences [10, 11]. It is further suggested that one cannot separate people's experiences from the context in which they have those experiences, thus, the nursing student's experiences regarding clinical support within the PHC clinical learning context [11]. The study setting was within one of the three North-West University campuses and focused on one school of the School of Nursing Science (SONS), of the two campuses that provides nursing education. The population included all nursing students from 1^st^ to 4^th^year level who were sampled using a purposive sampling technique. The sample size of this study, which was determined by data saturation, included 12 nursing students (all African, males and females), who complied with the following criteria: 1^st^ to 4^th^ year nursing student from the School of Nursing Science at the North West University; exposed to clinical practice and management of TB/HIV while in clinical practice. The researchers invited nursing students who were at the nursing building to take part in the study and collected data until saturation was reached. This meant that the researcher continued the research until no new information emerged [12]. Data were collected using an individual, unstructured, in-depth interview held during November 2016 in their own private rooms, each taking between 20-50 minutes, allowing the interviewers to access the deeper meaning of the nursing student's responses.

Two central open-ended questions were posed to the nursing students, thus, "what are the experiences of nursing students regarding clinical support in the management of TB/HIV patients and how can clinical support be strengthened and promoted among nursing students in the clinical environment?" The questions were then followed by probing [13]. The researchers also used field notes that contained information such as non-verbal cues and other gestures observed from nursing students. Data were transcribed verbatim. The researchers interpreted and gave meaning of the data to be able to gain understanding of the study [13]. Furthermore, data were organised, cleaned and analysed using Atlas TI. The Notice-Collect-Think (NCT) analysis was followed which involved two-phase analysis, namely descriptive level and conceptual level analysis. Descriptive level analysis involved exploring data and reading through the data to notice the recurrent logos category in the first stage [14]. This involved listening to the audio tapes, transcribing, reading through transcripts and coding until the researchers could no longer notice new codes, which led to second stage coding. The researchers continued with the coding and validated the code list [14]. Permission to conduct the study was obtained from the School of Nursing Sciences. Voluntary participation was ensured by allowing nursing students to participate voluntarily after signing an informed consent form. Furthermore, nursing students were informed that if they choose to withdraw from the study, they would be granted permission freely. Privacy was maintained through interviewing nursing students in their own rooms. Collected data were stored in a safe place which could only be accessed by the researchers. No names of the nursing students were used anywhere in the study as they were referred to by participant numbers e.g. participant 1, participant 2.

## Results

The participant's experiences regarding clinical support in TB and HIV management in PHC facilities were laid together using four themes as main headings with sub-themes under each. Table 1 reflects on the experiences of nursing students regarding clinical support in TB and HIV management in PHC facilities and measures to strengthen and promote clinical support. As indicated in Table 1, four themes emerged from the conducted interview, namely factors inhibiting clinical support towards nursing students in TB and HIV management; outcomes of poor clinical support in TB and HIV management; nursing students' desired outcomes through clinical support; and proposed strategies to promote and strengthen clinical support.

**Table 1:** Themes and subthemes of nursing students’ experiences regarding clinical support in TB and HIV management in PHC facilities

Themes	Sub-themes
Factors inhibiting clinical support in TB and HIV Management	Shortage of PNs
	Lack of accompaniment
	Fear of managing TB and HIV patients
	Inadequate preceptorship in TB and HIV management
	Less exposure to clinical services
	Negative attitude of PNs
	Lack of confidence among nursing students
Incompetence of nursing students	Inability to integrate TB and HIV theory into practice
Outcomes of clinical support in TB and HIV Management	Become a competent TB and HIV nurse Ability to integrate TB and HIV theory into practice
Promotion of clinical support	Strengthened Occupational Health and safety learning
	Provision of knowledge regarding PEP and infection control
	Increased allocation/placement time in PHC facilities
	Regular supervision/accompaniment by preceptors/lecturers
	Appointed clinical PN for students in each facility

**Theme 1: factors inhibiting clinical support towards nursing students in TB and HIV management:**factors inhibiting clinical support can be explained as the characteristics that deprive nursing students or create a wall between nursing students and the support from the nurses, preceptors, mentors, supervisors or lecturers when they are supposed to provide necessary backing in clinical facilities. The following factors were raised as inhibitors of clinical support towards nursing students, thus, shortage of PNs; lack of accompaniment; fear of managing TB and HIV patients; inadequate preceptorship in TB and HIV management; less exposure to clinical services; negative attitudes of PNs and lack of confidence among nursing students.

**Shortage of PNs:** it became evident that most nursing students do not receive adequate support in the clinical setting; this was verbalised by nursing students indicating that they do not receive adequate guidance and supervision in the facilities because of the shortage of professional nurses. This also prevented nursing students from knowing or understanding if they are providing proper TB/HIV management as they have no one to correct or applaud them. A nursing student verbalised that: *"Due to shortage of staff (sic), it brings about inadequate support to nursing student which makes us (Nursing Student) to work without supervision and guidance, and we cannot even determine if we are doing the wrong or right procedure"* (P8, Male, 25)

**Lack of accompaniment:** nursing students indicated that they are placed at the clinical setting with only a set of objectives, without any clinical accompaniment or support received from preceptors or module facilitators. This is serious, given that clinical accompaniment is a necessity and requirement of clinical teaching as nursing students need to be provided with clinical orientation and demonstrations before they can engage in any clinical skills that are set for them to practice. A nursing student indicated that: *"The university just send the set clinical objectives without coming to give guidance on how to practice them and without coming to give us (the nursing students) support."*(P6, Female, 22).

**Fear of managing TB and HIV patients:** nursing students are also faced with occupational exposure to HIV as well as TB during their clinical placement, given the procedures they are expected to conduct, such as blood collection via needle pricks. This exposure increases nursing students' fear for contagion or managing TB and HIV patients in which they need supervision and guidance. Clinical facilities sometimes lack the necessary resources they can use to protect themselves with, e.g. gloves and masks. A nursing student indicated that: *"one of the objectives of 2^nd^ year level is to do vein puncture, so we also practice it on people infected with HIV because they do sometimes come for blood collection and so injuries that some of our colleagues have experienced, lead us to fear to manage TB and HIV patients."* (P6, female, 22).

**Inadequate preceptorship in TB and HIV management:** the findings of this study also highlighted that there is a decline in clinical support as there are no longer active preceptors, as before. This reduction in preceptors, increases the level of support nursing students require to consume throughout their clinical learning. This was verbalised by a nursing student specifying that: *"There is no clinical support from the university because during my 1^st^ year there were preceptors of which now the preceptorship programme is no longer as active which leave us (nursing students) not receiving adequate clinical support."*(P8, Male, 26).

**Less exposure to clinical services:** nursing students who are pursuing a bachelor's degree tend to have less time to attend clinical services than those doing a diploma and they become less familiar with the ward environment and procedures done in the wards. Nursing students become incompetent in the management of TB and HIV due to less time spent in the clinical setting according to their placement. A nursing student reported that: *"During our 2^nd^ year, we only go to (the) clinical setting twice a week which is not enough for us to familiarise ourselves with the management of TB/HIV. (Sic) Give us time to learn and gain more experience regarding management of TB/HIV."* (P6, female, 22).

**Negative attitude of PNs:** PNs tend to have > negative attitude towards nursing students doing bachelor's degrees because they believe that they can work independently without any supervision and guidance which then promotes incompetency because they are not supported and guided during the practice of the clinical procedures. This was verbalised by a nursing student in the following way: *"There was a PN who had (a) negative attitude towards teaching us on (the) management of TB and when we asked her why she is not helping us, she said that we should know all procedures when we come for clinical placements so that we can be corrected."* (P1, Female, 21). Another student added: *"She (the Professional Nurse) told me to do something (and leave what I was doing) because I didn't know what I was doing, and I felt angry, sad and disappointed, because she did that in front of patients and I didn't feel right after that"* (P9, Male, 20).

**Lack of confidence among nursing students:** lack of confidence among nursing students to perform the tasks that are given to them, simply mean that, the students have the ability to do the task, however, this process is inhibited by the PNs' attitudes toward them even when they are able to do the task. This further reduces their confidence, as indicated by a nursing student stating that: *"Thorough clinical support helps us to integrate theory and practice and also build our self-confidence and it encourages us to continue wanting to learn more and do what is expected of us"* (P7, Female, 23)

**Theme 2: incompetence of nursing students:** incompetence of nursing students was brought about by the inability to perform the given tasks or inability to show competence with regard to the task or skill that they have been taught.

**Inability to integrate TB and HIV theory into practice:** most nursing students have difficulties in integrating theory to practice in the clinical setting. There are an increasing number of patients who need management of TB and HIV, so students end up setting aside their set objectives, as they are seen to be there to fill or compliment staff shortage gaps in order to push clinical work and end up unable to integrate what they learned theoretically into practice, as they don't get the chance to practice. A nursing student indicated that: *"(The) high number of patients and shortage of PNs which lead nursing students to work without supervision, guidance and accompaniment during management of TB and HIV patients in the clinical setting causes confusion to us because the theory which is taught in class needs to be applied in practice, but without supervision of the PNs, we are not sure whether we are doing the right or wrong procedures."* (P9, Male, 23). Another nursing student added: *"Theory should be given to nursing students prior (to) their clinical placement in order to integrate theory and practice at the clinical setting."* (P8, Male, 25).

**Theme 3: outcomes of clinical support in TB and HIV management:** this theme described the outcomes of clinical support which were inclusive of becoming a competent TB and HIV nurse; developing confidence in attending to TB and HIV patients; and the ability to integrate TB and HIV theory into practice.

**Becoming a competent TB and HIV nurse:** support and guidance provided to nursing students during clinical placement allows them to be able to do the work independently, even without supervision and to become competent, because they will be guided and corrected on mistakes they make when performing procedures. This also provides them with the ability to supervise other junior nurses as they feel competent and able to provide such clinical support. *"Being supported as (a) 3^rd^ year student allows me to become competent in a way that I will be able to guide and supervise the junior levels."* (P3, female, 22).

**Ability to integrate TB and HIV theory into practice:** support from the PNs and NEI allows nursing students to be able to integrate theory and practice because they will be supported and guided throughout all the processes. For nursing students to become competent they should be able to integrate theory and practice through supervision and guidance. *"Preceptors (from NEI) must collaborate with PNs/facilities, so that they teach students how to integrate what is being practiced, by following newly reviewed policies, rules and guidelines of facilities and Department of Health"* (P4, Female, 25).

**Theme 4: promotion of clinical support:** promotion of clinical support can be explained as coming up with strategies that the NEI or clinical facilities can use in order to enhance the clinical support of nursing students.

Strengthened Occupational Health and Safety learning: Policies must be strictly formulated, and preventive measures should be provided and promoted specifically for nursing students in order to ensure and promote safety of students during their clinical placement-these policies must be enforced in the NEI and also at the clinical facilities. It was verbalized that: *"It should be stressed that there should be a way where students are initially educated about OHS (Occupational Health and Safety), possible injuries during clinical practice, prevention of injuries and what to do after the incidence (reporting the incidence and the follow up there after), e.g. after touching blood or pricking yourself accidentally."* (P1, female, 21).

**Provision of knowledge regarding post exposure prophylaxis (PEP) and infection control:** the availability of PEP should be compulsory in all facilities because in some cases students would be struggling to get it and go from one facility to another after an incident. Sharp containers and other waste management policies must be regularly reviewed, and students informed on how to dispose waste and the importance of infection control, in order to protect themselves against contracting infections. A nursing student uttered that:*"We came across situation where students struggle alone to get PEP and had to deal with stress alone"* (P1, female, 21). Another nursing student verbalized that: *"Pre-registration must ensure that nursing students are being immunised for diseases, as a means to prevent diseases."* (P3, female, 23).

**Increased time for clinical allocation/placement in PHC facilities:** increased allocation/placement time in PHC facilities implies that the time allocated for the nursing students' stay in the clinical settings/ PHC facilities should be extend because they will then be able to learn more in the clinics during their longer stay. *"Give us time to learn and gain more experience regarding management of TB and HIV."* (P10, female, 22).

**Regular supervision/accompaniment by preceptors or lecturers:** preceptorship must be improved to ensure that strict supervision is provided and maintained, because most nursing students claimed that there is support from some of, and not all PNs, but support from NEI is very limited because preceptors does not come to the clinical placement facilities very often, or they don't come at all. A nursing student indicated that: *"They must make sure that they visit clinical learning settings for nursing students regularly in order to ensure adequate supervision and what is taught in class or theory is integrated practically at the clinical setting, because it is totally different as there is lack of clinical support with regard to the management of TB and HIV patients at the PHC setting, from both NEI and clinical placement."* (P1 female, 21).

**Appointment of PN in each facility:** there must be an appointed PN by NEI specifically for nursing students in each facility, whose responsibility is purely to guide and supervise students during their clinical allocation as nurses are faced with increased workload and they cannot completely provide sufficient supervision to nursing students. Some participants felt that they are being neglected by some of the PNs, and if there is an appointed PN as a preceptor, the set learning outcomes in clinical practice can be achieved. *"There should be a nurse appointed who is responsible for guiding and supporting students regarding their set learning objectives, the appointed PNs should be well equipped and trained to be able to provide support."* (P8, female, 23).

## Discussion

The study highlighted multiple factors that inhibit clinical support towards nursing students in TB/HIV management. The challenge of shortage of nurses had been identified as a major challenge in South Africa. Furthermore, this challenge is also experienced in many other countries like Ghana [15]. There is a critical shortage of PNs in South Africa and given the rise in TB/HIV infection, the increase in need for TB/HIV care rises sharply [16]. However, on the other side, it is clear that the Nursing Act of 2005 [17] provides for empowerment of PNs and midwives to assist, guide and support nursing students throughout their training, with the aim of developing competent, independent nurse practitioners by creating a conducive environment for learning and facilities where nursing students can obtain clinical exposure and learning. Yet, this had been proven to also pose a challenge to these PNs, given the imbalance of work between patient care and providing assistance, guidance and support to nursing students [2]. Due to the shortage of nurses, nursing students end up filling the gaps in order to push clinical work and not fulfilling their set objectives from NEI, and this deprives them to learn in clinical facilities and inhibits the support meant for them. However, it should be noted that nursing students may not be familiar with the guidelines and policies being used in the management of TB/HIV as there are new guidelines being introduced every now and then, hence nursing students need support from PNs to help them understand and familiarize themselves with those. The shortage of nurses often has an impact on the effectiveness of clinical accompaniment. Clinical accompaniment is a purposeful activity aimed at enabling a student to overcome their need for help and support, furthermore, it is an essential and interrelated function of clinical learning that enable the integration of theory and practice [18]. Inability to provide accompanist by the NEI and the clinical facilities where the students are placed, inhibits the chances of nursing students to develop and perform tasks without any inability or fear as most students indicated that they are sometimes afraid to manage PLWH.

Universal precautionary measures are extremely important and need to be emphasized and highlighted to all the nursing students at this level to reduce or curb occupational exposures to HIV. Nurses' exposure to HIV is a serious problem and has an impact in the provision of care towards PLWH, as nurses continually care for PLWH in settings where universal precautions cannot be sustained because of a lack of resources [16]. In addition, such emotions in nursing students, together with their clinical learning, may negatively affect their performance [4]. Nursing students become fearful and confused towards managing TB and HIV patients, given their fear of getting infected while providing care or practicing their clinical skills. This is closely linked to poor or inadequate preceptorship. There is a strong need for increment of preceptors to improve clinical support with regard to TB and HIV management as the School of Nursing Sciences cannot accomplish this alone. In addition, collaboration with the advanced practice clinical nurses, especially those who specialise in TB and HIV management within the Department of Health and other agencies, is indispensable in the provision of clinical support. Thus, nursing students need clinical learning facilitators who are available full time to ensure that clinical learning is guaranteed among the nursing students at all levels. It is also emphasized that clinical preceptors augment students' access to patients and clinical settings, clinical skill development, role acquisition, socialization, and professional transition into the world of advanced practice [19]. Furthermore, it had also been indicated that between the clinical learning environment and the classroom, a theory-practical gap may exist and to reimburse for this gap, clinical preceptors are employed, thus clinical support would be strengthened and promoted [20]. When there are clinical preceptors, the nursing students become relaxed and are able to show their incapability and the preceptors assist or coach them in a way that will develop their skills and competence. The preceptors' availability also helps the nursing students, as they are able to ask any questions they may have regarding anything they have come across at the health facility and are able to get the relevant answers, in a way students are able to put theory into practice and thereby closing the theory-practice gap. However, nursing students are not fully exposed to the clinical learning environment.

Nursing has a practical component and to ensure that the newly registered nurse is competent in the clinical environment, the nursing student must receive enough exposure in this environment. According to the South African Nursing Act (33 of 2005), the nursing student must work a minimum of 4000 hours in the clinical environment as part of a four-year integrated programme. Exposure to all the clinical fields of nursing is necessary to reach the particular outcomes as prescribed by the South African Nursing Council. Clinical Placement in a specific clinical environment allows students to gain experience of providing care to patients and assisting them to reach their long-and short-term goals [21]. Less exposure to the clinical facilities inhibits the chances of the nursing students to learn and be competent in a number of required skills and be exposed to clinical learning, and this is enlightened better by their inability to manage TB and HIV while they are at the clinical facilities. There are poor interpersonal relationships between PNs and nursing students. This is explained by the PNs' unwillingness to work with nursing students, promoting and creating a negative clinical practice environment [22]. Negative attitude of PNs as portrayed by the nursing students in this study, may also be seen as one of the reasons for the nursing students to end up being so incompetent when they complete their degree, as they tend to fall prey to the negative attitude of PNs as they deem them special and well informed and nursing students are always left alone without any sort of supervision. Furthermore, there are also PNs who lack the patience to actually teach or demonstrate nursing skills or procedures to the nursing students as they feel that it is time consuming and their routine becomes slower. Hence, they would rather strive to push and finish the routine care and not provide desired clinical teaching and learning to the students which had been shown in this study as a contributory factor to the incompetence of the nursing students. Clinical skill performance is reported to be the most influential source of self-confidence [23]. This means that self-confidence has been seen as a key component toward effective clinical performance, and it should be emphasized that confident students are more likely to become more effective nurses [23] and that, at the end, provides and promote quality nursing care. Recently, the academic simulation laboratory is heavily utilized to allow students to practice clinical skills in a controlled environment. The academic simulation laboratory setting serves the dual purposes of developing competency prior to practicing in the clinical setting and developing confidence, as skills are practiced in a safe environment where no harm can befall the student or patient. It was found that preparatory education in the way of integrated skills-based teaching, assisted students in acquiring confidence prior to clinical placement [23].

Lack of support in the health facilities by the PNs often lead to lack of confidence as the nursing student may not be sure if they are performing the clinical procedures correctly, which has the potential to lead to self-doubt. Confidence and ability go hand in hand, when one is being supported practically and clinically as one learn and practice; the ability to develop and perform the learned skills and confidence is enhanced. Evidence suggest that there is an excessive gap in integrating theory to practice which has been a concern in nursing education for a very long time and had negative impact towards students' in learning clinical skills [4]. Through guidance and support, nursing students would be able to integrate theory and practice as they would be supported and guided throughout their practice. In order to become a competent TB and HIV nurse practitioner, student nurses need to be guided and supervised thoroughly throughout their nursing training. Supervision of nursing students in clinical practice plays a significant role in the nursing profession, as it has an influence on the students' learning and skills development [4]. Nursing students become competent when they are corrected on their mistakes, and this then allows performing certain tasks like managing TB and HIV patients, to become more natural as they are supported, and guidance is provided where and when it is needed. Nurse's competence is based on the knowledge and skill they are taught during their pre-services nursing training. Nursing training is a combination of both theoretical and practical learning experiences that enables nursing students to acquire knowledge and develop skills needed to provide nursing care. Nursing education is composed of two complementary parts: theoretical training and clinical/practical training. The major part of nursing education is carried out in a clinical environment [24]. The nursing course emphasises critical thinking, problem solving, decision making and clinical skills, enabling nursing students to correlate theory and practice to reach educational outcomes as well as meet the needs of the community [25]. Integration of theory and practice is important because it allows one to be able to manage TB and HIV thoroughly and it enables nursing students to develop confidence and competency in the provision of such management. Nursing students are constantly in contact with patients and this exposes them to infections and thus require proper protective measures to reduce their risk of acquisition of disease or injury [26]. The occupational health and safety should be taken seriously as mostly infected-related injuries occur while managing TB and HIV patients so having a proper orientation to occupational health and safety would allow nursing students to be able to protect themselves and to utilise the available right channels to report the exposures, should they get exposed.

Effective measures to prevent nursing students from contracting infections through occupational exposure to blood, include immunization against HBV, eliminating unnecessary infections, implementing universal precautions, eliminating needle recapping, disposing of the sharps into a sharp container immediately after use and to educate nursing students about vaccination against diseases [27]. The provision of occupational health and safety in the clinical learning environment should be emphasized because the failure to do so exposes the nursing students to contagion and they become too anxious, to the point that they even fail to manage TB and HIV patients while they are in the clinical facilities. Literature indicates that five placement areas are obligatory: surgical nursing, medical nursing, mental health care, home care and nursing homes. Students must be assigned to any three of these areas for at least eight weeks and the other two for at least six weeks, and they study for 30 hours/week in the clinical area. These placements are defined as supervised practice. In supervised practice, the students are preceptored by a registered nurse [28]. The time allocated to the nursing students, especially the ones at the university, is less than that of students at the nursing colleges and thus inhibits the nursing students' ability to learn more at the facility and be able to develop their skills, confidence and competence. Preceptorship occurs as part of the regular clinical working hours. Senior students are assigned to experienced nurses who guide, act as role models for and support the students on a one-to-one basis. Preceptorship facilitates the development of the preceptees' clinical knowledge and skills as well as their attainment of professional competencies, to ensure they are fit to enter professional practice after they have concluded their supervised clinical practice [29]. Furthermore, preceptorship experiences for nursing students expose them to the knowledge and expertise of nurses working in a complex practical environment and the nurse preceptors facilitate learning and build nursing students' confidence [30]. Regular supervision of nursing students will enable the students to up their learning abilities and to become more inquisitive to learn even when learning may be difficult to them. The primary responsibility of the PN is the delivery of the highest standards of nursing care to patients. In addition, as professionals, they also have a responsibility to support the learning of other nurses, but due to imbalance of work, it is better to appoint a nurse to educate nursing students [31]. Appointment of a PN in each facility where the NEI places their students will also help when the preceptors who are appointed for clinical support, are not able to come to the clinics to provide clinical support to nursing students.

## Conclusion

The study was conducted with the aim of exploring the experiences of nursing students regarding clinical support in the management of TB and HIV in the PHC facilities. The study highlighted that there is inadequate support provided to nursing students during their clinical or practical placement. In addition, there is also a marked dearth of literature regarding clinical support of nursing students in the management of TB and HIV in the PHC context. Unavailability of policies which governs the clinical support of nursing students, also explain the uncertainty of who is responsible for the clinical support or accompaniment of nursing students when they are in the clinical facilities. It is recommended that NEI can strengthen and promote clinical support of nursing students through the development of clinical support policies, increasing supervision of nursing students in PHC facilities and appointment of a PN in each facility where nursing students are being placed for clinical learning in order to produce quality, healthy, skilful, confident and competent nurses.

### What is known about this topic

- There are multiple litigations among newly qualified nurses due to incompetence and lack of confidence in their caregiving role, mainly in the management of TB/HIV;
- Nursing students' fear of managing TB/HIV in the primary healthcare settings.

### What this study adds

- The study provides the experiences of student nurses regarding their lack of clinical support during their clinical learning and practice that negatively affects their competence and confidence through their study period;
- The study also highlights the need to strengthen clinical support within the NEI and the clinical learning settings as a means to promote the production of competent and confident newly qualified nurses.

## Competing interests

The authors declare no competing interests.
